# Cytokine gene polymorphisms implicated in the pathogenesis of *Plasmodium falciparum* infection outcome

**DOI:** 10.3389/fimmu.2024.1285411

**Published:** 2024-02-09

**Authors:** Selorm Philip Segbefia, Diana Asema Asandem, Linda Eva Amoah, Kwadwo Asamoah Kusi

**Affiliations:** ^1^ Department of Immunology, Noguchi Memorial Institute for Medical Research, College of Health Sciences, University of Ghana, Accra, Ghana; ^2^ Department of Molecular Medicine, School of Medicine and Dentistry, College of Science, Kwame Nkrumah University of Science and Technology (KNUST), Kumasi, Ghana; ^3^ Department of Virology, Noguchi Memorial Institute for Medical Research, College of Health Sciences, University of Ghana, Accra, Ghana; ^4^ West African Centre for Cell Biology of Infectious Pathogens, Department of Biochemistry, Cell and Molecular Biology, College of Basic and Applied Sciences, University of Ghana, Accra, Ghana

**Keywords:** cytokines, gene polymorphisms, plasmodium, immune response, pathogenesis

## Abstract

Cytokines play a critical role in the immune mechanisms involved in fighting infections including malaria. Polymorphisms in cytokine genes may affect immune responses during an infection with *Plasmodium* parasites and immunization outcomes during routine administration of malaria vaccines. These polymorphisms can increase or reduce susceptibility to this deadly infection, and this may affect the physiologically needed balance between anti-inflammatory and pro-inflammatory cytokines. The purpose of this review is to present an overview of the effect of selected cytokine gene polymorphisms on immune responses against malaria.

## Introduction

Globally, malaria is still one of the most prevalent parasitoses. The World Health Organization’s (WHO) 2022 World Malaria Report revealed that 95% of the estimated 247 million cases and 96% of malaria deaths globally occurred in Africa (1). The organisms that cause this disease are from the genus *Plasmodium*. These parasites are transmitted to a susceptible host when an infective female *Anopheles* mosquito takes a blood meal. Four different *Plasmodium* species infect humans, namely *P. falciparum, P. ovale, P. malariae*, and *P. vivax*. While *P. vivax* is the most widespread *Plasmodium* species globally, *P. falciparum* is the most prevalent and clinically dangerous, and is predominantly found in Africa, accounting for 99.7% of estimated global clinical malaria cases (1). *Plasmodium ovale* is further divided into two sub-species; *P. o. curtisi* and *P. o. wallikeri* (2). Aside from the typical human parasites, a number of simian parasites have also been recently found to cause human infections. *Plasmodium knowlesi* (3–5), *P. cynomolgi* (5–8) *P. inui*, *P. coatneyi*, and *P. simiovale* (6) are all semian parasites that have shown zoonotic potential and infect humans.

Currently, malaria is treated using artemisinin-based combination therapy (ACT), and the implementation of other transmission-blocking interventions like indoor residual spraying, chemoprevention in pregnant women and children, and long-lasting insecticidal nets usage (9). The use of vaccines is also effective in the prevention of malaria. It has been shown that the RTS,S, R21 and attenuated sporozoite candidates are capable of preventing clinical disease to some extent (10–13).

Naturally, the body fights this infection using both innate and adaptive immune mechanisms. A component of the innate immune mechanism, being the first line of action against infection, uses Pattern Recognition Receptors (PRRs) on the surfaces of inflammatory cells to recognize Pathogen Associated Molecular Patterns (PAMPs). *Plasmodium*-associated PAMPs such as haemozoin, parasite DNA, and glycosylphosphatidylinositol are recognised by Toll-like receptors (the main ones involved in the pathogenesis of plasmodia infections) (14), RIG-I-like, scavenger receptors (e.g., CD204 and CD 36), and NOD-like receptors. This results in a cascade of events that lead to the production of various cytokines required for the clearance of the parasites and also triggers the adaptive immune arm (15–18).

Vaccines work mainly through the adaptive mechanism of the immune system. This involves the production of antibodies by activated B lymphocytes (plasma cells) and the production of cytolytic factors by activated CD8+ T lymphocytes or their stimulation to undertake direct cytotoxic activity. Both mechanisms are achieved through the help of CD4+ T cells that provide signals through cytokines and chemokines (19).

Cytokines play a critical role in the immune mechanisms involved in fighting infections, including malaria. They act as messengers that help immune cells to communicate with one another in a concerted effort to clear infected Red Blood Cells (RBCs). Responses elicited by activated CD8+ T cells occur through the secretion of effector cytokines such as Interferon gamma (IFN-γ) (20). Memory CD8+ T cells are also maintained by cytokines such as IL-7 and IL-15 (21). The physiologic levels of these molecules within the immune compartment can therefore influence effective communication between the various immune cells. One significant factor that can affect cytokine/chemokine production levels is polymorphisms within the genes that encode for these molecules. Cytokine gene polymorphisms have been reported to affect cytokine production and, consequently, the effectiveness of an immune response to any invading pathogen (22). A meta analysis by Cui, Sun (23) revealed that polymorphisms in the IL-4 may affect Hepatitis B Virus (HBV) vaccination outcome. This implies that polymorphisms in cytokine genes may affect immune responses during an infection with *Plasmodium* parasites and immunization outcomes during routine administration of malaria vaccines. The purpose of this review is to present an overview of the effects of cytokine gene polymorphisms on immune responses to, and pathogenesis of malaria.

## Cytokines

Lymphocytes, haematopoietic, and inflammatory cells communicate with each other to effectively mount and coordinate a robust immune response. Soluble factors called cytokines act as messengers between these cells. They are a group of modulatory glycoproteins produced mainly by T helper cells, macrophages, dendritic cells, though they can be secreted by all nucleated cells (immune and non-immune cells (24–26).

Cytokines are grouped into six (6) families: interferons, transforming growth factor beta, haematopoietins, interleukins, tumour necrosis factor, and chemokines. This classification is based on the type of receptors they bind to and their three-dimensional structures (24). However, based on function, they can be grouped into five: interleukins, tumour necrosis factor, chemokines, interferons, and colony-stimulating factors (26). They play a crucial role in the immune system’s ability to recognize and respond to pathogens.

## Role of cytokines at different malaria parasite growth stages in the host

### Skin

**Figure 1:**
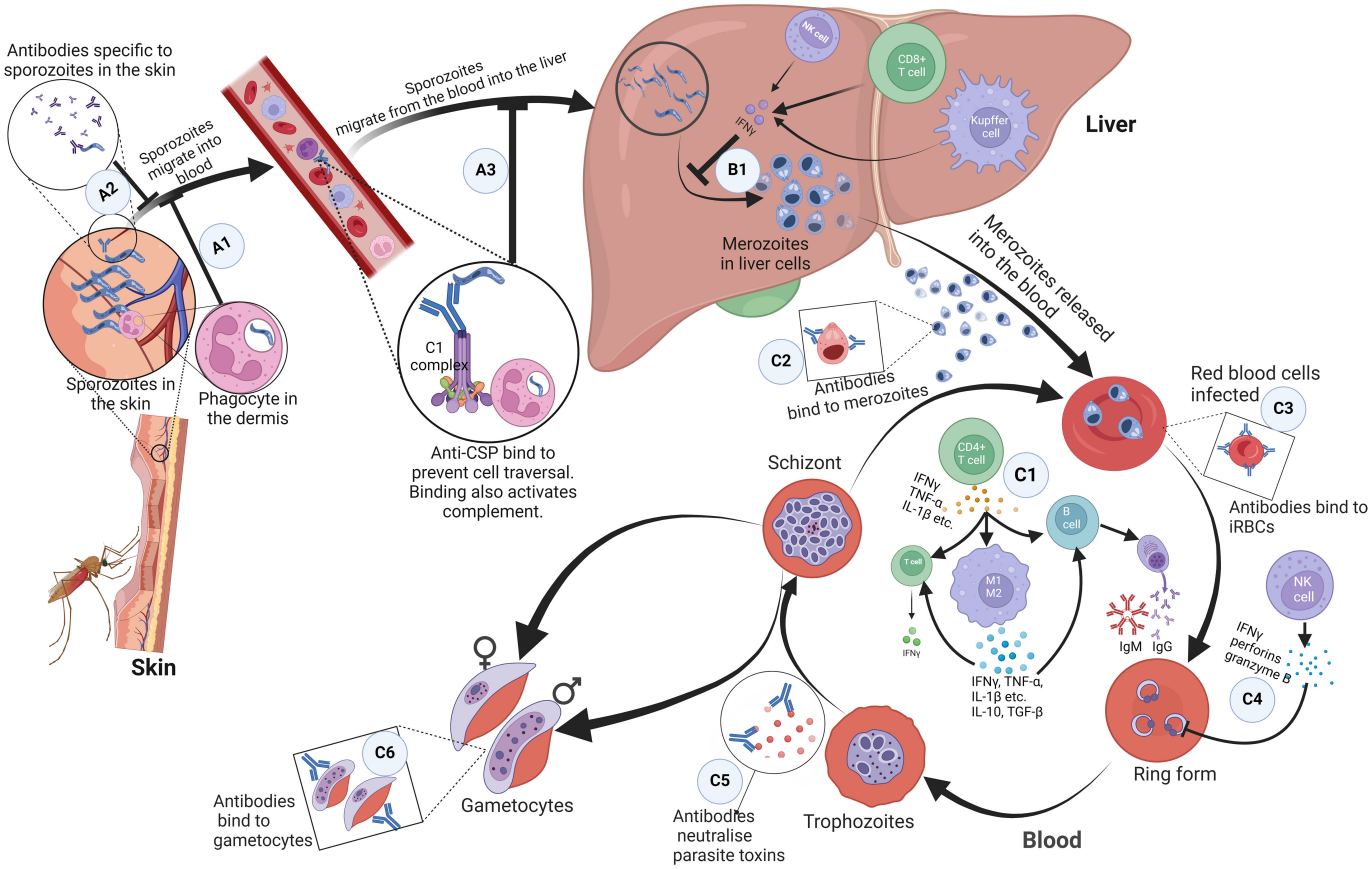
Immune response mechanisms to malaria parasites at different stages in the host. **(A1)** Antibodies bind to inoculated sporozoites and prevent them from entering the blood circulation. **(A2)** Phagocytes present in the dermis recognise and phagocytose sporozoites. **(A3)** Antibodies against free sporozoites bind and neutralise proteins required for cell traversal and invasion such as CSP. The binding of these antibodies also activates complement fixation, phagocytosis, and lysis by cytotoxic cells. **(B1)** NK cells, Kupffer cells, and CD4+ dependent CD8+ T cells that produce interferon-γ upon parasite recognition, kill intrahepatic parasites through an antibody-dependent cell-mediated mechanism. **(C1)** Proinflammatory cytokines produced by CD4+ T helper cells activate macrophages, B cell clones and T cells. Macrophages differentiate into pro-inflammatory and anti-inflammatory M1 and M2 phenotypes respectively. These activated macrophages produced cytokines; pro-inflammatory cytokines kill malaria parasites directly. Together, the pro and anti-inflammatory cytokines activate T cells and specific B cell clones. **(C2, C3)** Antibodies bind to and coat the surface of infected erythrocytes and merozoites. This prevents their adhesion to the endothelium and the uptake and invasion of erythrocytes. It also enhances their phagocytosis and clearance. **(C4)** Interferon-gamma, perforins, and granzyme B produced by NK cells kill parasites in infected erythrocytes. **(C5)** Antibodies neutralize parasite toxins to reduce excessive inflammation. **(C6)** Antibodies bind to gametocytes which are killed through complement-mediated lysis and this prevents sequestration and maturation of gametocytes in the host. ,CSP, circumsporozoite protein; NK, Natural Killer. Created with BioRender.com.

The skin acts as the first protective barrier to most infections, including malaria (27, 28). When sporozoites are carried past the epidermis of the skin by the mosquito’s proboscis, antibodies bind to the inoculated sporozoites and inhibit their mobility, thereby preventing them from entering the bloodstream (**Figure 1 A1**) (29–31). Additionally, CD8+ T cells are primed by dendritic cells from lymph nodes draining into this site of the skin, consequently boosting immune response in the liver (32). Phagocytes can also recognise and phagocytose sporozoites in the dermis (**Figure 1 A2**) (33). If the sporozoites are not cleared, they find their way into blood circulation.

### Pre-erythrocytic stage

In the blood, to prevent the invasion of hepatocytes and initiate the pre-erythrocytic cycle, antibodies against free sporozoites bind and neutralise proteins required for cell traversal and invasion, such as the circumsporozoite protein (CSP). The binding of these antibodies also activates complement fixation, phagocytosis, and lysis by cytotoxic cells (**Figure 1 A3**) (34, 35). In the liver, Natural Killer (NK) cells, NK T cells, Kupffer cells, and CD4+ dependent CD8+ T cells that produce interferon-*γ* upon parasite recognition, kill intrahepatic parasites through an antibody-dependent cell-mediated mechanism (**Figure 1 B1**). Other cells, such as the Gamma Delta (*γδ*) T cells also kill intrahepatic parasites through secretion of type I interferons and IFN-*γ* (36–40). There is growing evidence of the effectiveness of CD8+ T cells that secrete IFN- and granzyme B at killing infected hepatocytes (20, 41). The failure of these mechanisms leads sporozoites to undergo sporogony in the hepatocytes and develop into merozoites.

### Erythrocytic stage

Merozoites egress from hepatocytes to start the erythrocytic stage. This stage is characterised by the signs and symptoms associated with clinical malaria. It is associated with the production of proinflammatory cytokines by CD4+ T helper cells, which are needed to activate macrophages, specific B cell clones, and other cells (**Figure 1 C1**) (36–39, 42, 43). Humoral (antibody) and T-cell responses are also important for the control of merozoites and intraerythrocytic parasites, respectively. Antibodies bind to and coat the surface of infected erythrocytes and merozoites, targeting antigens such as the erythrocyte binding antigen (44), to prevent their adhesion to the endothelium and the uptake and invasion of erythrocytes respectively (**Figures 1 C2, C3**). This enhances their phagocytosis and clearance (34, 45). However, this is only possible during the limited window when merozoites break out of a fully developed schizont and before they infect the next RBC. To achieve this, Tumour Necrosis Factor (TNF) and IFN-γ activate macrophages to phagocytose and/or kill infected erythrocytes and merozoites (**Figure 1 C1**). Antibodies also bind to gametocytes, which are killed through complement-mediated lysis, preventing the sequestration and maturation of gametocytes in the host (**Figure 1 C6**) (46). Antibodies and complement taken up in the blood meal also mediate the lysis of gametocytes, preventing fertilization and further development of the parasite in the mosquito (34). To reduce excessive inflammation, antibodies neutralize parasite toxins by binding to glycosylphosphatidylinositol (**Figure 1 C5**). Interferon-gamma, perforins, and granzyme B produced by NK cells kill parasites in infected erythrocytes (**Figure 1 C4**) (36–39, 42, 43).

Inferentially, pro-inflammatory cytokines kill malaria parasites directly. They also act in ‘concert’ with other anti-inflammatory cytokines to activate T cells and specific B cell clones to produce cytolytic chemicals, IgG (47) and IgM (48, 49) respectively, needed for a robust immune response (**Figure 1 C1**).

### Immunopathologic role of cytokines in *P. falciparum* infection

Cytokines are highly relevant for the development of immunity against malaria parasites, regardless of the human stage of the parasite. However, excessive production of certain cytokines is also linked to malaria immunopathology and directly related to some of the clinical symptoms. Additionally, cytokine polymorphisms can affect the activity, quantity, and timing of cytokine production, influencing susceptibility to and severity of malaria. Polymorphisms in the TNF-α, IL-1β, IL-4, IL-10, IL-13, IL-17, IL-18, IL-22 and CXCL8 genes have been shown to affect their function during malaria infections. It is, therefore, essential to identify cytokines and polymorphisms that play critical roles in the protection and/or pathophysiology of malaria. This information can help to make informed decisions regarding malaria vaccination programmes. The following cytokines have been selected for this review because they have been directly implicated in either protection or susceptibility to malaria.

### Interferon gamma

Interferon-γ (IFN-γ), a 20-25 kDa protein, is one of the many pleiotropic cytokines that play a pivotal role in coordinating innate and adaptive immunity. The gene encoding IFN-γ is located on the short arm of chromosome 12 and codes for a 166 amino acids peptide (50, 51). The production of IFN-γ is vital to initiate an immune response against *Plasmodia*, both at the pre-erythrocytic and erythrocytic stages (43, 52). Early production prevents the progression from uncomplicated malaria to severe or life-threatening infection (53). Though conflicting reports exist on the role of IFN-γ in the pathogenesis of malaria, a deeper look at these findings reveals that these differences may be influenced by host and parasite factors. Concomitant increases in IFN-γ and IL-6, IL-2, IL-5, IL-12 levels were observed during *Plasmodium* infection (54). This helps differentiate patients with mild cases of malaria from those with severe forms of the disease. However, when high levels occur together with high IL-12 levels, it discriminates severe forms from asymptomatic infection. Nevertheless, persistently high levels may cause inhibitory effects on CD4+ T cells, hence leading to immune suppression (54).

### Tumour necrosis factor-alpha

The TNF-α gene (3 kb), made up of four exons and three introns, is located on chromosome 6 (55). Structurally, TNF-α, a 17 kDa is a homotrimer protein consisting of 157 amino acids. This is a product of the precursor made up of 233 amino acid residues (26 kDa) (56). Tumour Necrosis Factor is involved in multiple inflammatory conditions and immune responses, playing an important role in the pathogenesis of many infectious diseases, including *P. falciparum* malaria (57). Normal levels of TNF-α are essential for parasite clearance. This was observed in a Ghanaian population where asymptomatic individuals had higher levels compared to uninfected individuals (52). Parasite clearance is believed to occur through a mechanism in which TNF-α increases the phagocytosis of *Plasmodium* parasites (15, 58). However, high levels are linked to severe forms of malaria (57, 59) and this may explain why it has been reported to be associated with the development of Cerebral Malaria (CM), a complication of severe malaria. This association may be due to the induction of the expression of adhesion molecules for the attachment of parasites on endothelial cells of the cerebrum (43). However, these reports still need further research to ascertain findings. Cruz, Wu (60) showed that TNF-α lowers parasite counts by increasing intracellular calcium levels. Also, in synergy with IL-1β, they activate transcription factors necessary for the production of IL-6 (61), which helps to clear the infection.

Polymorphisms in the promoter (15, 62) and non-coding regions of the TNF-α gene have been linked to an increased risk of *Plasmodium* infection. The rs673 Single Nucleotide Polymorphism (SNP) is associated with repeated mild infections with *Plasmodium* while the rs1800629, rs673, and rs361525 SNPs are linked to symptomatic infections (63). Also, in an Indian population, the homozygote SNPs (rs1799964 and rs1800630) in the TNF-α enhancer region lead to higher levels of plasma TNF-α, while the rare allele of the SNP (rs41297589) and the heterozygote forms of the SNPs (rs1799964, rs1800630, rs1799964, rs361525, and rs1799724) increased the risk of getting severe forms of malaria (57, 64). The TNF-α SNP genotypes rs1800629 (-308G/A) GG, GA, AA, G and A alleles were not associated with malaria among populations from different countries around the world (62, 65). However, the TNF-α SNP genotypes for rs1799964 (-1031C) (TT, TC, CC), rs1800750 (-376) (AG, GG, AA), rs1800629 (-308) (AG, GG, AA) rs36152 (-238) (AG, AA, GG) rs3093662 (+851) (AG, AA, GG) and rs1800630 (-863A) (CC, CA, AA) alleles (66), as well as homozygotes for the TNF enhancer haplotype CACGG (rs1799964 (-1031T>C); rs1800630 (-863C>A); rs1801274 (exon4 G>A); rs1800629 (-308); rs361525 (-238); rs41297589 (-76) across Indian populations) (57), and rs1800750 (-376) GAG (67), correlated with enhanced plasma TNF-α levels in both patients and controls. Significantly higher TNF-α levels were observed in patients with severe malaria. Minor alleles of rs1799964 (T) and rs1800630 SNPs were associated with increased susceptibility to severe malaria (57, 67).

### Transforming growth factor-beta

Transforming growth factor beta (TGF-β) is a T cell inhibitory cytokine that plays a vital role in maintaining a balanced and tolerant immune system by suppressing the growth and activity of numerous immune system elements. It is a central cytokine in the differentiation of both Treg and Th17 cells (68). Out of the three isoforms, TGF-β1, is the most relevant from the standpoint of immune regulation (69). The genes for the three isoforms- TGF-β1, β2, and β3- are located on chromosomes 19q13.2, 1, and 14, respectively. These genes code for inactive precursors which are then activated to form a 25 kDa protein (70). Transforming growth factor-beta regulates the function of other important cells in malaria immunity: Dendritic cells, which produce IL-4 in the acute phase of malaria (71), T-helper 17 and T regulatory cells (68, 72). It is a more potent T-regulatory cytokine than IL-10 in malaria immunity (15), and higher levels of TGF-β are inversely proportional to severe forms of malaria (73). A proportional increase in TGF-β and TNF-a, IL-10, and IL-1β, predicts a proportional increase in severe forms of malaria, especially in CM patients (54). A finely tuned balance between TGF-β and IL-6 appears to be pivotal and possibly determines the outcome of *Plasmodium* infection (68). Genotypes and alleles reported in the TGF-β1 gene include rs1800469 (-509C/T) (CC, CT, TT, C and T); however, no association with malaria was found (65).

### Interleukin 1-beta

IL-1 was among the first cytokines to be discovered, in addition to IL-18 (24, 74). Initially known to be the pyrogen that causes fever, it was later identified as the lymphocyte-activating factor. From that point, it was shown to initiate and mediate myriad immunological processes, such as prostaglandin synthesis, neutrophil influx and activation, T-cell activation and cytokine production, B-cell activation and antibody production, fever, and fibroblast proliferation and collagen production (74). There are two isoforms, IL-1α and IL-1β, produced by distinct genes but binding to the same receptor. IL-1α is expressed constitutively by a majority of cell types and does not need cleavage to become active, though its biological activity can be enhanced through Calpain II processing. It is released from cells during cell death as it is a membrane-bound molecule, hence signals the immune system of tissue damage (24).

On the other hand, IL-1β is expressed by myeloid immune cells as a 31 kDa zymogen (pro-IL-1β), which is made active through cleavage. The gene encoding this 269 amino acids protein is located on chromosome 2q14.1 (75). Its secretion enhances the recruitment of neutrophils and the differentiation of T-cells into Th17 cells. In effect, the secretion of IL-1β signals the recognition of a threat by the innate immune cells. It is believed to be involved in the first line of immune response mounted against pathogens including Plasmodia (76, 77). It is also needed for the activation of transcription factors necessary for the production of IL-6 (61). However, increased levels are associated with CM (54).

Single nucleotide polymorphisms in the IL-1 promoter region predispose children to severe malarial anaemia as a result of increased levels of IL-1β. Point mutations at position 31 and 511 in the promoter regions, where there is a change to cytosine and adenine, respectively, increase the risk of severe malaria due to reducing the production of circulating IL-1β (78). Similar findings were reported by Walley, Aucan (79) in Gambia and Brazil (64). However, the mutant allele AA/AG in IL-1β (rs1143634) was shown to protect against *P. falciparum* infections (80). In terms of humoral response, the rs16944 SNP was linked to the production of more antibodies in response to a *P. vivax* circumsporozoite protein (81). The SNP genotypes and alleles of IL-1β, rs16944 (-511 T/C) (TT, TC, CC, T and C), were not associated with malaria (65). However, in another study, the IL-1β SNPs, rs1143627 (-31 C/T) (CC, CT, and TT), rs16944 (-511 A/G) (AA, AG and GG) were associated with SMA especially the CA haplotype. The SNP, (-31T/-511A) (TA) haplotype causes increased production of IL-1β (78). Additionally, a study in The Gambia demonstrated significant associations between variation at IL-1β +3953 position (rs1143634 C/T) and susceptibility to clinical malaria (79).

### Interleukin 4

The IL-4 gene is located on chromosome 5q31.1 (82). The role of IL-4 in human malaria has not been extensively studied. In animal models, it has been reported to increase the risk of severe malaria. This may be due to its role in downregulating pro-inflammatory processes vital for the clearance of the parasites. In a study in Sudan, high levels were rather associated with hyperparasitaemia rather than the clinical severity of the disease (83). However, Wu, Brombacher (71) found that in the absence of IL-4, mice were protected against CM. Also, mice treated with IL-4 analogues were protected against the development of CM. This intervention reduces the expression of CXCL10, CXCR3, and adhesion molecule (LFA-1) by T cells, increases the production of IgM, and downregulates the cytolytic activity of cytotoxic T cells. This significantly reduces the motility and the infiltration ability of CD8+ T cells into the brain, hence reducing brain damage (84).

There are not many reports on the SNPs in this gene in relation to malaria. However, in a Brazilian population, point mutations in the third intron and at position 33 (rs2070874) and 589 (rs2243250) in the promoter regions, did not show any difference in antibody levels, plasma IL-4 levels, and peripheral parasitaemia (85). A study among Cameroonians showed similar results in the IL-4 SNP, rs2243250 (-589 T/C) (CT, TT, CC) (66).

### Interleukin 6

The interleukin-6 (IL-6) gene is located on chromosome 7p21 and encodes a precursor peptide of 212 amino acids. The active peptide is made up of 184 amino acids (61, 86). The core protein is ∼20 kDa, but glycosylation accounts for the size of 21–26 kDa of natural IL-6 (61). It is a multifunctional cytokine produced by diverse cell types and plays an important role in various biological responses (87). The production of IL-6 is not limited solely to immune cells; endothelial, mesenchymal cells, fibroblast, and adipocytes, are also sources of IL-6. Among the many pleiotropic effects of IL-6, it is also important in the differentiation of naïve B and T cells to other effector subsets- an essential requisite for adaptive immunity (61, 88, 89). In an experimental cerebral malaria model, IL-6 was vital in the expansion of myeloid-derived suppressor cells to a pro-inflammatory phenotype. These cells were associated with malaria immunopathogenesis in humans, where they suppress the CD4+ T cell activity (90). Other studies show that IL-6 acts in concert with TNF-α and other inflammatory mediators in the clearance of malaria parasites (91, 92) but a meta-analysis by Wilairatana, Mala (88) showed that although IL-6 may be a marker of *Plasmodium* infection, increased levels are associated with hyperparasitaemia. This has been corroborated by the results of Mbengue, Niang (59), Frimpong, Amponsah (52) and Prakash, Fesel (54), where high levels are associated with severe forms of malaria. This may be a physiological response by the body to clear the parasites, increasing the levels of IL-6 in response to increasing parasitaemia.

### Interleukin 10

Interleukin 10 is one of the major T-regulatory cytokines, apart from TGF-β and IL-35. It suppresses antigen presentation, T cell activation, and proliferation mediated by dendritic cells and macrophages and inhibits the production of proinflammatory cytokines (53, 93). The 5.1kb pairs gene that encodes this protein is located on chromosome 1q31-q32 (93, 94). Its levels are very essential in maintaining a balance between pro- and anti-inflammatory cytokines in the control of *Plasmodium* infections (95). Essentially, IL-10 enhances the humoral immune response to *Plasmodium* infection (96) but inhibits cell-mediated responses (96). High levels have been associated with susceptibility to malaria due to its role in downregulating the pro-inflammatory processes needed to clear the parasite (43, 53, 54). On the contrary, the results of Mbengue, Niang (59) show that IL-10 levels are the same in both asymptomatic and severe malaria. This can be due to the fact that similar levels are needed to achieve the effect needed, hence levels may not be significantly different in either asymptomatic or severe cases.

Variations in cytokine promoter sequences, such as the IL-10 promoter, may specific transcription factor recognition sites and consequently affect transcriptional activation and cytokine production (66). There are conflicting reports on the rs1800896, a promoter region SNP in IL-10. Natama, Rovira-Vallbona (80) reported an increased risk for malaria infection, while the results from da Silva Santos, Clark (67) and Apinjoh, Anchang-Kimbi (66) showed a decreased risk of cerebral malaria. However, the IL10 rs3024500 SNP has been found to increase the risk for severe forms of malaria (66). In infants, it was found to decrease the risk of infection (94). The report by (66) showed that only the rs1800890 SNP was associated with IL10 production, although studies indicated associations between different SNPs and variations in IL-10 production. This suggests that rs1800890 may upregulate IL-10 transcription, with the heterozygotes potentially providing a selective advantage, as elevated IL-10 levels can down-regulate proinflammatory cytokines such as TNFα, offering protection against severe malaria. Individuals with the IL-10 rs1800890 AT genotype exhibited higher IL-10 plasma levels compared to homozygotes (66). The genotypes and alleles of IL-10 SNPs, (rs1800896) (-1082A/G), (AA, AG, GG, A and G alleles), rs1800871 (-819T/C), (TT, TC, CC, T and C alleles), reported among populations worldwide showed no association with malaria (65). However, the IL-10 SNP rs1800896 (-1082 A/G), GCT haplotype, and the rs1800871 (-819T/C), and rs1800872 (-338 592A/C) were associated with a reduced risk of malaria symptoms (67). Plasma IL-10 levels strongly correlated with the heterozygous AT genotype of IL-10 rs1800890 SNP. The AA and TT genotypes of IL-10 rs1800890 had lower plasma IL10 levels compared to their AT counterparts. Additionally, the IL-10 SNP genotypes; rs3024500 (AG, GG, AA), rs1800896 (TT, CT, CC), rs1800890 (TT, AT, AA), rs1800896 (TT, CT, CC) and rs3024500 (AA, GA, GG) were associated with CM, while the rs1800896 SNP genotypes (CT, CC, TT) were linked to hyperpyrexia (66). However, rs3024500 and rs1800896 were associated with altered CM risk in another study (66). The -1082G/-819C/-592C (GCC) haplotype was associated with protection against SMA and increased IL-10 production. Although none of the other haplotypes were significantly associated with susceptibility, individuals with the -1082A/-819T/-592A (ATA) had an increased risk of SMA and reduced circulating IL-10 levels (97).

### Interleukin 12

Interleukin-12 was the twelfth cytokine to be identified in 1989. It was the first to be identified in the IL-12 family comprising of IL-12, IL-23, IL-27, and IL-35. It consists of two subunits, a smaller p35 monomer (35 kDa α-chain) and a larger p40 monomer (40 kDa β-chain), encoded on chromosomes 3q25.33 and 5, respectively. The co-expression of the smaller and larger subunits results in the formation of the biologically active p70 heterodimer (98, 99). Interleukin-12 secretion mainly regulates the production of IFN-γ through the differentiation and polarisation of naïve T cells to Th1 phenotype. This helps in mounting an immune response against *Plasmodium* species (100). Recent findings show that it acts synergistically with IL-18 to modulate γδ T cells (101), which control parasitaemia via an antigen receptor-mediated degranulation and the phagocytosis of antibody-coated infected RBCs (102). They regulate the function of γδ T cells by increasing the expression of T cell immunoglobulin domain and mucin domain-containing protein 3 (TIM3) and hence decreasing the risk of symptomatic malaria (101). Levels of IL-12 have been reported to differentiate CM and Severe Malaria Anaemia (SMA) from Mild Malaria (MM) (54).

### Interleukin 13

Interleukin-13 (IL-13) is a 33-amino acid protein encoded by a 4.6 kb gene located on chromosome 5q31 (103). It belongs to the Th2 family of cytokines, which includes IL-4 and IL-5. It is primarily produced by CD4 T cells, adaptive effector cells involved in allergic asthma. However, in humans, innate immune cells like eosinophils, basophils, mast cells, natural killer (NK) cells, and NK T cells have also been observed to be capable of producing IL-13 (104–106). In malaria, the rs20541 polymorphisms is linked to high risk of developing cerebral malaria (107). Ohashi, Naka (108) earlier reported an association of IL-13 variation in the promoter region (1055 C>T) with reduced susceptibility to malaria in Thailand. Additionally, the IL-13 46457 SNP genotypes, rs20541 (CT, CC, TT), were found to be associated with *Plasmodium* infection (66).

### Interleukin 17

Interleukin 17 (IL-17) is a distinct family of cytokines comprising at least six members (A–F) that share unique characteristics. It is an ~18 kDa glycoprotein composed of 163 amino acids encoded by a gene located on chromosome 6 (109–111). Interleukin 17 is secreted by CD4 T helper 17 and CD8 cytotoxic T17 cells, which typically function as a pro-inflammatory cytokine (15, 112). High levels have been reported during the acute phase of malaria (113) and are involved in erythrocyte remodelling; a mechanism thought to protect against this infection (114).

Apinjoh, Anchang-Kimbi (115) showed that polymorphisms in the IL-17 gene (rs6780995) are associated with hyperpyrexia, and they suggested a possible link with uncomplicated malaria.

### Interleukin 18

Interleukin 18 is a pro-inflammatory cytokine encoded by a gene located on chromosome 11q23.1 (116). Initially thought to stimulate T cells, NK cells, and activated macrophages to produce IFN-γ (117, 118), it was hence named IFN-γ-inducing factor. IL-18 is first synthesised as a 193 amino acids zymogen of 24 kDa, which is cleaved to an active 18 kDa protein (116). It belongs to the IL-1 family of cytokines that promotes Th1 responses. When IL-18 is present without IL-12 but in the presence of IL-2, it activates NK cells, CD4+ NKT cells, and existing Th1 cells to generate IL-3, IL-9, and IL-13. Moreover, in combination with IL-3, IL-18 triggers mast cells and basophils to produce IL-4, IL-13, and various chemical mediators (119). It helps protect against infectious organisms, including *Plasmodia*, through the induction of IFN-γ in a murine models (100). It acts together with IL-12 in the innate immune mechanism against *Plasmodium* infection (101). In individuals suffering from *P. falciparum* malaria, the levels of IL-18 in the bloodstream are elevated. When comparing malaria patients based on the severity of their condition- non-complicated, severe, and cerebral malaria - increased IL-18 levels were observed across all three groups. Among severe malaria cases, IL-18 tended to remain high throughout the course of the disease. Furthermore, a noteworthy correlation was found between the level of IL-18 in severe malaria patients and the degree of parasitaemia (120, 121).

Polymorphisms in the promoter and 3` untranslated regions have been linked to *Plasmodium* infection. The rs5744292 SNP was associated with parasitaemia in all age groups while alleles of rs360714 and rs544354 pose a risk of infection in younger children (122). A study on the sequence variations of the human IL18 promoter region revealed several SNP genotypes: rs1946519 (-656 G/T), rs1946518 (-607 C/A), rs187238 (-137 G/C), rs360718 (+113 T/G), and rs360717 (+127 C/T) (122). Out of the several SNPs identified, the SNP genotypes rs5744292 (AA, AG, GG, A and G alleles) and rs544354 (GG, AG, AA, G and A alleles),as well as rs360714 (GG) and rs7106524 (AG), were found to be significantly associated with increased parasite density in infected patients. Moreover, several haplotypes were found to have a significant association with parasitaemia (122).

### Interleukin 21

The IL-21 gene is positioned on the human chromosome 4q26-q27, and the precursor and mature IL-21 polypeptide comprises 162 and 131 amino acid residues, respectively (123). Interleukin 21, a 15 kDa protein, plays a role in the synthesis of immunoglobulins, similar to IL-6 (59). Also, in response to activating signals, IL-21 stimulates the growth of mature B and T cells and facilitates the expansion of NK cell populations originating from the bone marrow (123). In mouse models, IL-21 deficiency resulted in higher parasite counts, affected B-cell responses, and subsequent production of immunoglobulins (124).

### Interleukin 22

Interleukin-22 (IL-22), initially referred to as IL-10-related T cell-derived inducible factor (IL-TIF), is a member of the IL-10 family of cytokines, produced mainly by activated Th1 cells (66). This cytokine family also comprises IL-10, IL-19, IL-20, IL-24, and IL-26 (125). The human IL22 gene is located at chromosome 12q15 (126). The IL22 gene contains an open reading frame of 537 base pairs, which encodes a 179-amino acid protein with a 79% similarity between mice and humans (127). The cytokine’s active and secreted form is a shorter protein, comprising 146 amino acids (128). Interleukin-22 is mainly produced by activated Th-17 cells, γδ T cells, NKT cells and newly described innate lymphoid cells which belongs to the IL-10 family of cytokines (125, 129). Available evidence implicates IL-22 in the pathogenesis of *Plasmodium* infection and vulnerability to severe malaria anaemia has been shown to decrease with increased IL-22 levels (130).

In a Saudi Arabian population, the IL-22 SNPs; rs2227481, rs2227513, and rs2227483 were reported to protect against *Plasmodium falciparum* infection. The G allele of the SNP rs2227513 is associated with increased levels of IL-22 (130). Koch, Rockett (129) showed that the IL22 SNPs coexisting together (+1394A and -708T) and (-708C and +1394A) were associated with protection and vulnerability, respectively, to severe malaria. This findings were similar to that of Apinjoh, Anchang-Kimbi (66). The IL-22 SNPs rs2227507 (+4583 C/T), rs1012356 (+2611 A/T), rs2227491 (+708 C/T), rs2227485 (-485 A/G), rs2227478 (-1394 A/G), were not associated with malaria (66). Observations from a Cameroonian population supported earlier studies in The Gambia, reporting an association of the IL22 + 708T allele with protection against severe anaemia (66). SNPs in the promoter region of the IL-22 gene, specifically rs2227476 and rs2227473, were associated with CM in both Nigerian and Malian children. Individuals carrying the aggravating T allele of rs2227473 produced significantly more IL-22 than those without this allele. Overall, these findings suggest that IL-22 is involved in the pathogenesis of CM (131).

### Chemokine ligand 8

Chemokine (C-X-C motif) ligand 8, the most potent human neutrophil-attracting inflammatory chemokine, coordinates the directional chemotaxis of leukocytes to inflammatory sites (132). Also known as IL-8 (118), located on chromosome 4 locus q12-4q21 (133–135). Its transcribed DNA yields a non-functional peptide of 99 amino acids, further cleaved into different shorter polypeptides of about 15 kDa (134–136). Neutrophils produce this chemokine during *Plasmodium* infection due to the production of histamine-releasing factors by the parasite. High levels have been reported in severe malaria patients (59).

A SNP at position 251, where an adenine replaces thymine in the promoter region, increases the susceptibility to complicated *Plasmodium* infection (65).

## Concluding remarks

**Figure 2:**
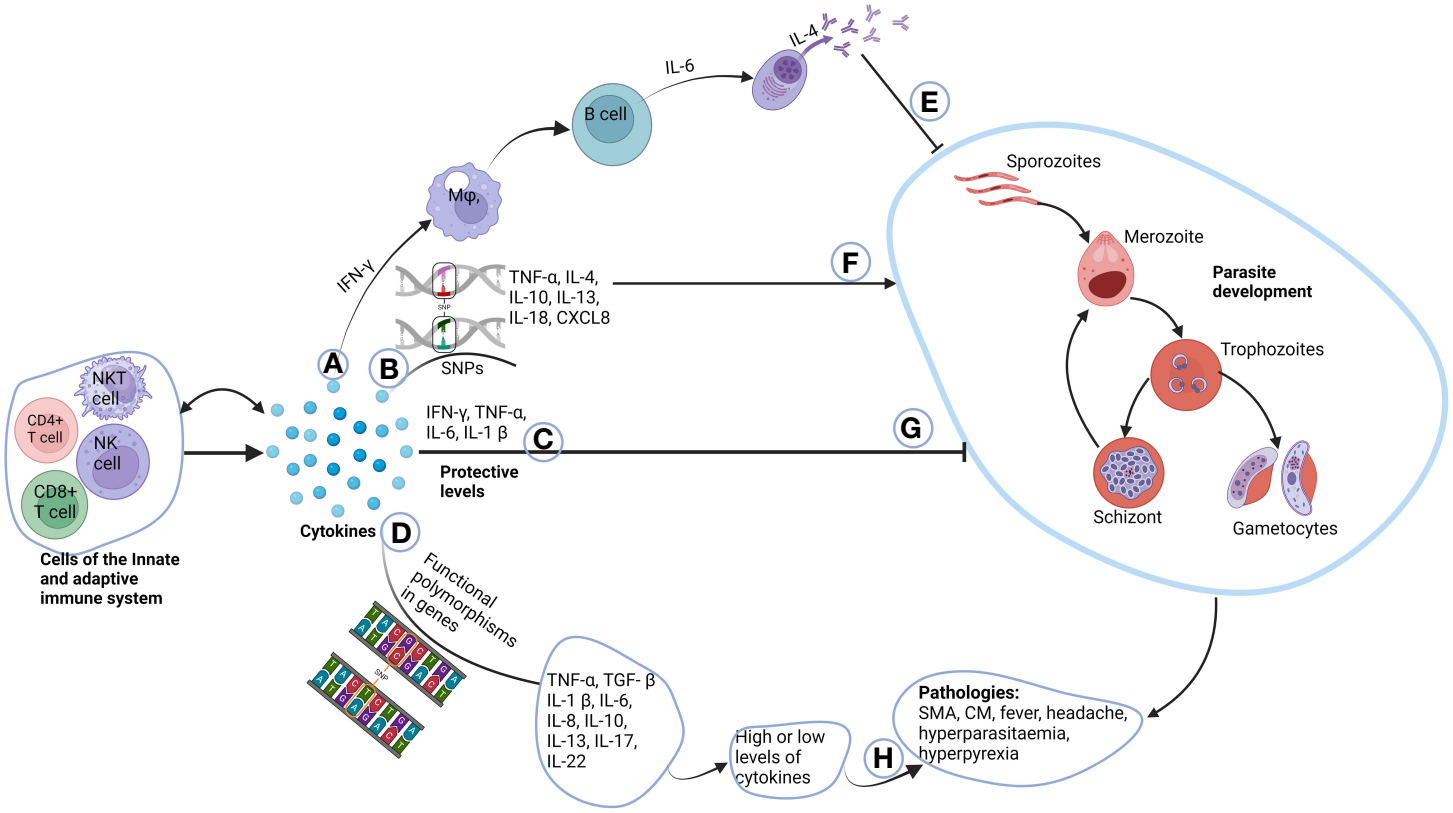
Role of cytokines in the pathogenesis of malaria. Cells of the innate and adaptive immune system produce cytokines, where some have autocrine function. **(A)** IFN-γ produced by T cells activate macrophages that in-turn activate B cells to differentiate to plasma cells through the help of IL-6. Plasma cells then produce immunoglobulins which can switch classes with the help of IL-4. **(E)** The immunoglobulins then inhibit parasite growth. **(B)** Unique functional polymorphisms in cytokine genes of TNF-α, IL-4, IL-10, IL-13, IL-18 and CXCL8 increases susceptibility to malaria parasite development **(F)**. **(C)** Normal plasma cytokines levels which are deemed to be protective, inhibit parasite development **(G)**. **(D)** Functional polymorphisms in TNF-α, TGF-β, IL-1β, IL-6, IL-10, IL-13, IL-17 and IL-22 either cause increased or decreased production of these cytokines. **(H)** This may account for some of the pathologies and complications associated with malaria. Created with BioRender.com.

This review has provided a comprehensive overview of the effects of selected cytokine gene polymorphisms on immune responses during malaria. It is evident that cytokines play a very significant role in both the protection against *Plasmodium falciparum* infection and the pathogenesis of malaria. Polymorphisms in the genes that produce these cytokines may affect the delicate balance needed between anti-inflammatory and pro-inflammatory cytokines, resulting in an increased or reduced susceptibility to this deadly infection (**Figure 2**). Moving forward, future investigations should consider employing advanced tools, such as whole-gene sequencing, to delve deeper into the biology of cytokines in the context of *Plasmodium falciparum* infection. This approach promises to unravel finer genetic nuances, offering insights that genotyping techniques alone might overlook. Considering the evolving landscape of infectious diseases, ongoing investigations in this field are crucial for advancing our ability to combat *Plasmodium falciparum* infection effectively.

## Author contributions

SS: Conceptualization, Writing – original draft, Writing – review & editing. DA: Writing – original draft, Writing – review & editing. LA: Supervision, Writing – original draft, Writing – review & editing. KK: Supervision, Writing – original draft, Writing – review & editing.
